# Reference Interval of Hemoglobin A1c and Influence of Hematological Parameters on Its Serum Concentration in Dogs

**DOI:** 10.1155/2020/7150901

**Published:** 2020-10-07

**Authors:** Stephan Neumann

**Affiliations:** Small Animal Clinic, Institute of Veterinary Medicine, University of Goettingen, Burckhardtweg 2, D-37077, Goettingen, Germany

## Abstract

HbA1c could be an alternative to fructosamine as a marker for glucose levels over a longer period. In this study, we calculated a reference interval for HbA1c in dogs and investigated the correlation of HbA1c with hemoglobin and different hematological parameters. In total, 110 blood samples from dogs were investigated. Significant negative correlations were found between HbA1c and erythrocyte count, hemoglobin concentration, as well as hematocrit. There was a tendency in the red cell distribution width. No significant correlation was found in the reticulocyte number and the erythrocyte indices. In conclusion, there is an association of different blood parameters with the HbA1c concentration, which have to be considered for the interpretation of HbA1c.

## 1. Introduction

Diabetes mellitus is one of the most frequent endocrine diseases in dogs. Various studies have assessed the prevalence of diabetes mellitus which ranges between 0.3 and 1.3% [1–4]. The diagnosis of diabetes mellitus is based on compatible clinical signs and clinicopathological alterations such as hyperglycemia and glycosuria. However, blood glucose levels can vary widely, and measurement of a second parameter, which gives more information about the glucose level over a long period, is recommended.

Fructosamine gives an estimate of blood glucose concentration for the last 1–3 weeks because this is generally the half-life of albumin and other proteins that are bound with glucose [5, 6]. This parameter is commonly used in animals, whereas in humans, instead of fructosamine, hemoglobin A1c (HbA1c) is favored. HbA1c is part of the HbA fraction which can be divided into the subtypes HbA1a1, HbA1a2, HbA1b, and HbA1c [7]. Plasma glucose binds to HbA1c during the whole life of erythrocytes and reflects the plasma glucose levels over the lifetime of an erythrocyte, which is three months in many species, for example, dogs. In humans, measurement of HbA1c instead of fructosamine for the diagnosis and monitoring of diabetes mellitus has been recommended since 2010/2011 [8]. This is due to the better correlation of HBA1c with blood glucose levels and a longer reflection of the average blood glucose concentrations compared to fructosamine [9]. Recently, some studies have investigated the use of HbA1c in dogs with diabetes mellitus [10–12]. There are some advantages in measuring HbA1c instead of fructosamine in dogs, for example, fructosamine is produced in the liver; thus, diseases of the liver influence the fructosamine serum concentration [13]. Furthermore, fructosamine is influenced by hyperbilirubinemia, hyperlipidemia, and azotemia [6, 14]. Influences on the HbA1c serum concentration are rarely reported in veterinary medicine. However, because HbA1c is glycated hemoglobin, alterations in hemoglobin or erythrocyte concentrations and metabolisms may influence the HbA1c concentration. The aim of this study is to compare serum HbA1c concentrations with different hematological indices to see if there is an association of hemoglobin or erythrocytes with the HbA1c serum concentration, which have to be considered by the interpretation of HbA1c in dogs.

## 2. Materials and Methods

### 2.1. Animals

In total, 110 dogs of different breeds, sex, and age were included in this study. All dogs were patients at the Small Animal Clinic, University of Goettingen. The samples for this prospective study were collected between April and November 2019. In all cases, blood samples were taken for diagnostic reasons. Blood samples were collected from healthy dogs (*n* = 45) for routine examination or from nonhealthy dogs (*n* = 65) including cases of diabetes mellitus (*n* = 2) and anemia (*n* = 10). All procedures were according to the German law on animal welfare.

### 2.2. Blood Sampling and Measurements

Blood samples from all dogs were collected from the cephalic vein. Blood for hematology was collected using an EDTA tube (Fa. Sarstedt AG and Co, Nümbrecht, Germany) and blood for the measurement of fructosamine, HbA1c, and glucose was collected using a serum tube (Fa. Sarstedt AG and Co, Nümbrecht, Germany). Following collection, hematology parameters were investigated immediately using the ProCyte Dx Hematology Analyzer (IDEXX Laboratories Inc., One IDEXX Drive, Westbrook, Maine 04092, United States). The following parameters were investigated: hemoglobin (Hb), erythrocyte count (RBC), hematocrit (HCT), red cell distribution width (RDW), reticulocytes, mean corpuscular volume (MCV), mean corpuscular hemoglobin (MCH), and mean corpuscular hemoglobin concentration (MCHC).

Blood for the measurement of fructosamine, HbA1c, and glucose was centrifuged directly after sampling (3000 g/5 min) (Fa. Eppendorf AG, Hamburg, Germany), and the measurement was obtained directly after centrifugation. Fructosamine was measured by a clinical chemistry analyzer (Konelab 20 i; Fa. Thermo Fischer Scientific Inc., Dreieich, Germany) and a commercial kit, using a colorimetric method with nitro blue tetrazolium (NBT). HbA1c was also measured by a clinical chemistry analyzer (Konelab 20 i; Fa. Thermo Fischer Scientific Inc., Dreieich, Germany) and a commercial kit. The principle of the test is a turbidimetric inhibition immunoassay. HbA1c reacts with anti-HbA1c antibody and forms a soluble antigen-antibody complex. A polyhapten reacts with the excess anti-HbA1c antibodies and forms insoluble antibody-polyhapten complexes, which are determined turbidimetrically. The imprecision of the test as determined by intra-assay and inter-assay coefficient of variation is 2.8% and 1.6%, respectively.

### 2.3. Statistics

Statistical analysis was conducted using the program Prism 8 (GraphPad Software 2365 Northside Dr. Suite 560 San Diego, CA 92108, United States). The data were checked for normal distribution using the Kolmogorov–Smirnov test. For descriptive statistics, mean, minimum, and maximum were calculated. A linear regression analysis was used to assess the relationship between the different groups. A *p* value less than 0.05 was considered statistically significant. The reference range was calculated using the following formula: mean ± 1.96 × SD.

## 3. Results

### 3.1. Demographics

Different breeds of dogs were included without any marked breed prevalence. The age of the animals ranged between 0.5 and 14 years with a mean of 6.3 years. The sex distribution was almost equal with 55 female and 65 male dogs.

### 3.2. HbA1c

The level of HbA1c in all animals ranged between 6 and 43 mmol/mol and was normally distributed. Forty-five healthy dogs were used as controls. The range of HbA1c in this group was 17.1 ± 3.56 mmol/mol.

### 3.3. HbA1c and Hematological Parameters

**Figure 1:**
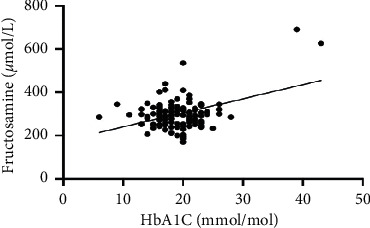
Relation between fructosamine and HbA1c (*r* = 0.175; *p* < 0.0001).

**Figure 2:**
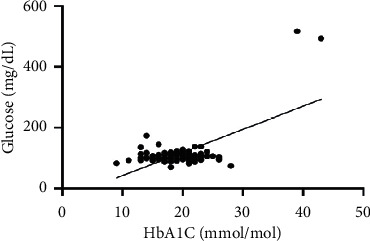
Relation between glucose and HbA1c (*r* = 0.386; *p*=0.0001).

**Figure 3:**
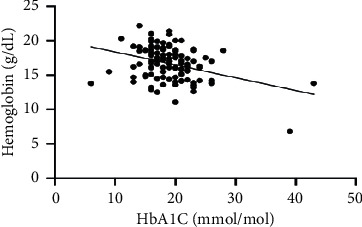
Relation between hemoglobin and HbA1C (*r* = 0.127; *p* < 0.0001).

**Figure 4:**
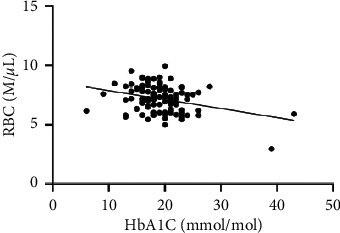
Relation between red blood cells and HbA1c (*r* = 0.104; *p* < 0.0004).

**Figure 5:**
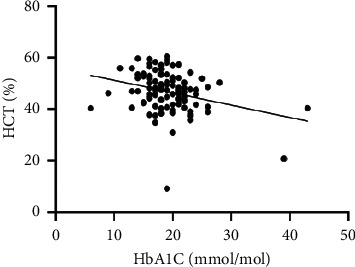
Relation between hematocrit and HbA1c (*r* = 0.083; *p*=0.0017).

**Figure 6:**
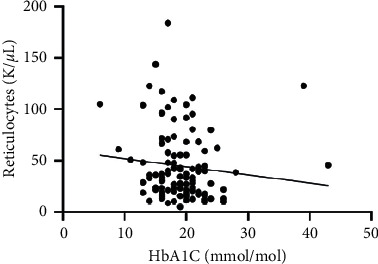
Relation between the reticulocyte number and HbA1c (*r* = 0.011; *p*=0.248).

**Figure 7:**
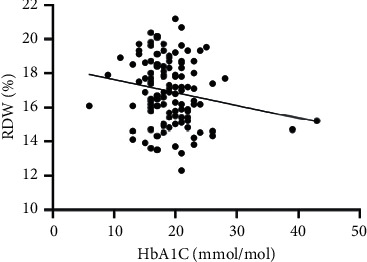
Relation between the red cell distribution width and HbA1c (*r* = 0.038; *p*=0.053).

**Figure 8:**
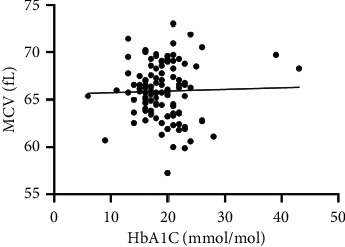
Relation between MCV and HbA1c (*r* = 0.0007; *p*=0.772).

**Figure 9:**
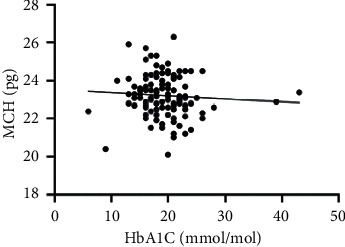
Relation between MCH and HbA1c (*r* = 0.0047; *p*=0.463).

**Figure 10:**
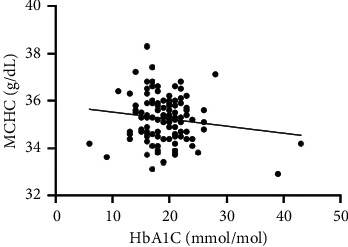
Relation between MCHC and HbA1c (*r* = 0.02; *p*=0.126).

As hematological parameters Hb, RBC, HCT, RDW, reticulocytes, MCV, MCH, and MCHC were measured (Table 1). There was a weak to moderate correlation between HbA1c and fructosamine as well as glucose in our study (*r* = 0.175; *p* < 0.0001; *r* = 0.386; *p* < 0.0001) (Figures 1 and 2). The correlation with hemoglobin was weak (*r* = 0.127; *p* < 0.0001) but negative (Figure 3). A similar correlation was observed between HbA1c and red blood cells (*r* = 0.104; *p*=0.0004) (Figure 4). A weak negative correlation existed between HbA1c and the hematocrit (*r* = 0.083; *p*=0.0017) (Figure 5). No significant correlation could be found to the number of reticulocytes (*r* = 0.011; *p*=0.248) (Figure 6) and the RDW (*r* = 0.038; *p*=0.053) (Figure 7), and finally, no significant correlation was found when HbA1c was compared with the erythrocyte indices (MCV *r* = 0.0007; *p*=0.772; MCH *r* = 0.004; *p*=0.463; MCHC *r* = 0.02; *p*=0.126) (Figures 8–10).

## 4. Discussion

Following its first description in 1955 [15], it was more than 50 years before HbA1c was recommended as the standard parameter for diabetes mellitus diagnosis and control in humans [8, 16]. One of the reasons for this long delay was associated with the establishment of a standard measurement procedure. Today, HbA1c is measured with the HPLC ion exchange method, the affinity chromatography, the enzymatic assay, or the immunoassay, and the results are given in percent or mmol/mol [10, 12, 16–18]. The formula used to transform percent into mmol/mol is as follows:

(1) $$\begin{array}{c} \text{HbA}1\text{c}\left(\text{\%}\right)=\left(\text{0.}09148\times \text{HbA}1\text{c}\left(\text{mmol/mol}\right)\right)+2\text{.}152\left[17\right]\text{.} \end{array}$$

From the described measurement methods, the immunoassay was the method we used for dogs in our study. Based on this method, reference intervals for HbA1c were calculated in humans as 20–42 mmol/mol [19] and in dogs as 5.3–20.7 mmol/mol [18] and 9.0–18.5 mmol/mol, respectively [10]. In this study, the HbA1c serum concentration of healthy controls ranged from 13.5–20.6 mmol/mol. Because the normal hemoglobin concentrations in humans and dogs are equal (8.0–11.0 mmol/l), the different reference intervals between both species cannot be explained by different hemoglobin concentrations. Comparing our HbA1c range in healthy controls with the other canine studies [10, 18], we found comparable upper limits, but a difference between the lower limits. We do not have any explanation for this difference because the inclusion criteria for reference dogs are equal in the different studies. For clinical purposes, just the upper limit is of importance [12], so the difference in the lower limits is without any clinical relevance.

Today, HbA1c is the gold standard for the assessment of serum glucose concentration over a longer period in human medicine [20]. In former times and in veterinary medicine, long-term hyperglycemia (1–3 weeks) is diagnosed by an elevated fructosamine concentration. Different human and veterinary studies show a significant correlation between HbA1c and fructosamine [11, 18, 20–22]. This was confirmed in our study. We found a weak to moderate correlation between HbA1c and fructosamine or glucose. Other studies showed a high correlation between HbA1c and fructosamine, but in these studies, more diabetic patients were considered, having an influence on the significance [11, 18].

Based on the physiology of HbA1c, an influence of different alterations in hemoglobin metabolism or erythrocytes on HbA1c is possible. Various studies have compared hematological parameters with HbA1c in humans, and inverse correlations with different parameters including hemoglobin and erythrocyte indices such as MVC were found [23–25]. In veterinary medicine, only a few studies have investigated HbA1c in cases of anemia and compared HbA1c with hematological parameters [14, 18]. Correlations between HbA1c and hematological parameters were inconsistent. In our study, we found a weak negative correlation between HbA1c and hemoglobin, the RBC, and the HCT. As HbA1c is part of hemoglobin, it is probable that alterations in hemoglobin or the RBC also induce HbA1c alterations. This was confirmed by the results of our study. In contrast to other studies, we could not confirm a relationship between HbA1c and erythrocyte indices. This is may be a consequence of a low number of anemic dogs in our study population (*n* = 10). In our study, there was a correlation tendency between HbA1c and RDW. This was confirmed by other studies, where HbA1c significantly correlated with RDW [26, 27]. The reason for this tendency is not clear because the RDW is a parameter of erythrocyte volume like MCV, which does not correlate with HbA1c. Furthermore, we also found no correlation between HbA1c and the reticulocyte count.

In one study, a combination of HbA1c and HCT was used to detect gestational diabetes mellitus in humans [28]. In our study, we could not confirm this observation as we did not include enough dogs with diabetes mellitus, but a significant correlation between HbA1c and HCT was observed.

## Figures and Tables

**Table 1:** Descriptive statistics of the hematological analytes.

	Hb (g/dl)	RBC (M/*μ*l)	HCT (%)	RDW (%)	Retic. (K/*μ*l)	MCV (fl)	MCH (pg)	MCHC (g/dl)
Min	6.8	2.97	9.1	12.3	4.9	57	20	32
Max	22.2	9.96	61.7	21.2	183	73	26	38
Mean	16.7	7.19	50.1	16.9	44	65	23	35

## Data Availability

The data used to support the findings of this study are available from the author upon request.
